# “If you do vasectomy and come back here weak, I will divorce you”: a qualitative study of community perceptions about vasectomy in Southern Ghana

**DOI:** 10.1186/1472-698X-14-16

**Published:** 2014-05-08

**Authors:** Philip Baba Adongo, Placide Tapsoba, James F Phillips, Philip Teg-Nefaah Tabong, Allison Stone, Emmanuel Kuffour, Selina F Esantsi, Patricia Akweongo

**Affiliations:** 1 Department of Social and Behavioural Sciences, School of Public Health, University of Ghana, P. O. Box LG 13, Accra, Ghana; 2 Population Council, 14B Ridge Road, Roman Ridge, Accra, Ghana; 3 Department of Population and Family Health, Mailman School of Public Health, Columbia University, 60 Haven Avenue, B-2, New York, NY 10032, USA; 4 Department of Epidemiology and Disease Control, School of Public Health, University of Ghana, P. O. Box LG 13, Accra, Ghana

**Keywords:** Vasectomy, Pregnancy, Family Planning, Male involvement, Southern Ghana

## Abstract

**Background:**

Male involvement in contraceptive use is increasingly becoming a global reproductive health issue. Vasectomy is one of the two male modern contraceptive methods espoused by the National Family Planning Policy in Ghana. Despite these advocacies, there are reports of low patronage of this method in Ghana. This study adhering to RATS guidelines on qualitative research therefore explored the social and cultural factors that may be affecting the low vasectomy uptake in Southern Ghana.

**Methods:**

The study was conducted in Sefwi Bibiani-Ahwiaso Bekwai (SBAB) District and Komenda-Edina-Eguafo-Abrem (KEEA) Municipal area in the Western and Central regions of Ghana respectively. Twelve Focus Group Discussions were held with both male and female community members. In-depth interviews were also carried out with Community Health Officers (CHOs), Community Health Volunteers (CHVs) and health managers at both the district and regional levels. The discussions and interviews were recorded, transcribed verbatim and analysed using Nvivo 10.

**Results:**

The study revealed that vasectomy was perceived as an act against God, which was punishable either by death or answerable on judgement day. Vasectomy was also perceived to be a form of castration, which can make men weak and incapable, thereby unable to satisfy their wives sexually, leading to marital conflicts. Women were more concerned about the negative effects of vasectomy on men. Cafalgin and panacin which are locally manufactured analgesics were perceived to have contraceptive abilities and therefore used by men as an alternative to modern contraceptive methods.

**Conclusions:**

Stigma and the misconceptions in the community may be accounting for the low vasectomy uptake in Ghana despite several advocacy strategies. Women were highly influential in a man's decision on vasectomy. This calls for the need to increase health education to demystify the misconceptions about vasectomy. Vasectomy-related campaign messages should target both men and women.

## Background

In the year 2013, the world population reached 7.2 billion and this high population growths are of global concern. Total fertility rate has been declining globally, however, the rate of decline is slow among less developed countries where the use of modern contraceptive methods is low which is about 31% among women of reproductive ages who are married or in union in 2013 [1]. Therefore, as a measure to control births, many countries make it a reproductive policy to promote the use of contraceptives in addition to advocacy to involve men in family planning issues. One strategy to foster male involvement in family planning is to give couples more contraceptive choices through the promotion of male-oriented methods such as vasectomy. Vasectomy is considered one of the most reliable family planning methods currently available with very low post-vasectomy pregnancy rates. These rates have been estimated to be between 0 - 2% [2].

Vasectomy is a safe, simple and effective method of family planning [3]. This procedure can be performed in low resource settings [4] and therefore could be an alternative to tubal ligation for women. Despite being a simple and safe procedure, some complications can emanate from the procedure. Common long-term complications from vasectomy are scrotal pain, with about 1% reporting pain that noticeably affects quality of life [5] and spontaneous recanalisation of the vas deferens that occurs in 0.03-1.2% after previous clearance of spermatozoa in the semen [6].

Presently in Ghana, the use of male condoms and vasectomy are the two main contraceptive methods available to males. The low level of post-vasectomy complications makes vasectomy one of the safest and reliable contraceptive methods available for males in Ghana. However, vasectomy remains one of the least known and least used family planning method. A retrospective review of 271 vasectomies performed between 1 January 2000 and 31 December 2009 in three healthcare facilities in Ghana revealed that, less than 0.5% of family planning clients opted for vasectomy in Ghana. The mean age of vasectomy acceptors was 40.7 years, and their average number of children was four [7]. Another study conducted in Ghana also showed that 39% of the people related vasectomy with castration and considered it a shameful procedure [8].

To increase the awareness and improve acceptance of vasectomy, the Ghana Health Service, the U.S. Agency for International Development (USAID) Mission in Ghana, and EngenderHealth (under its former cooperative agreement) collaborated on an initiative in the Accra and Kumasi metropolitan areas in 2003. This initiative adopted a combined strategy using site interventions that focused on quality of care and access (supply-side interventions) with effective and strategic interventions aimed at increasing public awareness on vasectomy (demand-side interventions). In 2004 and 2008 the ACQUIRE Project launched the first and second phases of projects on vasectomy respectively. The overall objectives of the 2004 and 2008 communications campaigns were to raise people’s awareness regarding vasectomy, to increase their awareness of the availability of the services, and to serve as a catalyst for men considering vasectomy [9]. Though the ACQUIRE projects have made some gains in creating the awareness on vasectomy in Ghana, it still remains a less patronized contraceptive method among couples. This study adhering to RATS guidelines on qualitative research was therefore designed to explore community perceptions about vasectomy and how these perceptions may be affecting vasectomy uptake in southern Ghana.

## Methods

### Ethical considerations

Ghana Health Service Ethics Committee approved the protocol for the study. All study respondents provided written or oral informed consent based on respondent’s preference. To those who gave oral consent, the consenting process was recorded digitally on a separate digital recorder at the beginning of the interview. The digital recorder containing the verbal consents given by respondents were kept separately from the ones used to record the interviews. They were further made to recommend an independent person to serve as a witness in the consenting process and the demographic data of the witnesses were documented. Personal identifiers and locator information were not collected, and any identifying information accidentally mentioned was removed from the text prior to analysis.

### Description of study area

The study was conducted in two districts, one in the Western Region and another in the Central Region in 2012. These districts were Sefwi Bibiani-Ahwiaso Bekwai (SBAB) district and Komenda-Edina-Eguafo-Abrem (KEEA) municipal area in the Western and Central regions respectively. The 2010 Population and Household Census indicated that the populations of these two districts were 123, 272 and 144, 705 respectively [10]. The main economic activity in the SBAB district is mining and subsistence farming whereas the main occupation in KEEA municipal is fishing. Both areas are fairly homogeneous with the Akan ethnic group forming about 80% of the population [10]. The districts are fairly served with health facilities and several functioning Community-based Health Planning and Service (CHPS) compounds.

### Selection of Focus Group Discussions (FGDs) participants

Twelve (12) FGDs were conducted, six in each of the districts: three for male community members and three for female community members. The female discussants were put into three categories. These categories were 18 to 24 years, 25 to 40 years and above 40 years. However, male respondents were grouped into two; those below 35 years of age and those who were 35 years or above. For each of the two male groupings, three FGDs were conducted making the total of six FGDs. Participants were married and had between 2–5 children. Semi-structured, open-ended data collection instruments were used in the study. The FGD guides covered areas such as knowledge and perception on vasectomy, local beliefs about the procedure, and ways to increase vasectomy acceptance. The FGD guides were translated from English to Akan using a back-to-back translation strategy. In this strategy, the FGD guide was first given to a language expert proficient in both English and Akan to translate the guide from English to Akan. The Akan version was subsequently given to another language expert to retranslate the guide back to English and this was compared to the original English version. Where there were discrepancies, these were resolved by the two language experts with a third language expert acting as a moderator. To ensure consistency of questions among multiple interviewers, the research assistants were trained. The training was a combination of theoretical training on how to conduct interviews and FGDs and mock interview sessions under the supervision of the researchers. All FGDs were conducted in Akan, the local dialect by native speakers. Respondents were asked the same questions in the same sequence; inductive probing on key responses was conducted. Each FGD consisted of eight discussants seated in a semi-circle with the moderator and note-taker in middle of the discussants. In all, 48 women and 48 men took part in the FGDs. Each FGD lasted for between 60–90 minutes. During the FGDs, each participant was given the opportunity to give his or her contribution to any theme raised before proceeding to another theme.

### Selection of the In-depth Interviews (IDIs) participants

Purposive sampling was used to recruit Community Health Volunteers (CHVs), Community Health Officers (CHOs), and Public Health Nurses (PHNs) for an in-depth interview for a provider’s perspective on vasectomy. In all 16 CHVs, 16 CHOs, and 20 PHNs were recruited equally distributed across the two study areas and interviewed. These were health workers engaged in the provision of reproductive health services at the community, district and regional levels.

### Data processing and analysis

All FGDs and IDIs were audiotaped using digital audio-recorders. Audiotaped data were transcribed into Microsoft Word for Windows. In select cases, the original words or phrases in Akan language were left in the transcripts. In addition, field notes were taken on paper. Transcripts were reviewed for obvious errors by both field staff and one of the investigators. Errors were corrected in the transcripts only after discussing the transcriptions with the interviewer/transcriber to ensure appropriate meaning. At least two independent persons carried out the transcription for a given FGD and IDI. A codebook was then developed, discussed and accepted by the researchers. The transcripts and field notes at this stage were converted into rich text format and imported into QSR Nvivo 10^©^ software. Nvivo is a software for textual analysis of large qualitative datasets. Guided by the objectives of the study, thematic content analysis was employed in the analysis of the data. A line-by-line coding of the dataset was done within Nvivo. This involved reading through responses of the participants and assigning codes to specific aspects of the responses. In assigning codes the researchers ensured that code names reflected specific word choices by respondents. At the beginning, the data were coded but not categorized (free nodes) since it was not very clear how they fit in the study and what the relations were between them. However, after the data analysis developed, the relationships between the codes became easier to see and the researchers started grouping them using tree codes (codes in a hierarchical mode). Afterwards, the codes were compared and in about 80% of the times the codes agreed. Where there were disagreement, they were discussed by the researchers to arrive at an appropriate meaning without compromising the original meaning. After finishing the coding process, the nodes in the node browser were reviewed to see the patterns that emerged on the community's perceptions on vasectomy from the study. Themes were subsequently formed from the patterns that emerged on perception regarding vasectomy and these are presented in the results.

## Results

### Spousal pressure

A wife’s disapproval emerged as a major factor that can influence a men’s intention to accept vasectomy or not. Disapproval from the women was due to the fact that vasectomy was perceived to make a man weak and incapable of offering sexually gratification to a woman. To community members, a good man should be able to perform his "sexual duties" very well but with the perception that vasectomy could reduce man's ability to perform this duty, it was perceived as unfavourable procedure for men who are concerned about giving their wives sexual gratification. It was believed that a woman who is unable to get the sexual satisfaction from the husband was likely to indulge in extramarital sex with other men to acquire sexual pleasure. Besides the sexual weakness, women also believed that vasectomy could result in physical weakness making the man less productive.

In a quick response, a man in a focus group discussion who had previously contemplated going in for vasectomy and decided to seek his wife’s opinion narrated the rebuttal he received from the wife “….If you go and do anything (vasectomy) and come back here weak, I will divorce you”. This refusal by women to allow their husbands to do vasectomy was also expressed by health managers who are service providers.

“…. *For instances where even men want to do the vasectomy*, *the women warned them against it*, *so you realize that it is the women who prevent the men from doing vasectomy*”- (A senior health manager in IDI).

There was however a more preference for female methods in the community and this emerged as a well-entrenched theme in both FGDs with males and females community members as well as IDIs with health care providers. Men perceived family planning as the responsibility of women. Men also expressed fear regarding the potential failure of condom to offer the desired protection and pain and risk associated with male sterilization.

"*The men in this community see family planning as a female matter*, *so it is difficult to talk to them about a procedure such as vasectomy*"- (IDI, Male CHV).

### Misconceptions and misinformation

Vasectomy was associated with many misconceptions and misinformation. Many respondents indicated they obtained much of the information on which they base their family planning decisions from sources within the community such as family members and friends. These individuals lack the knowledge to provide accurate and complete information for an informed choice. The result is that many of the information received by community members are inaccurate. Many respondents also indicated that they have never heard of or seen a vasectomy acceptor. The community members believe that a testimony by a vasectomy acceptor could help disabuse their minds and encourage them to accept vasectomy.

“…. *I have not yet seen any man who has done vasectomy*… *at least if we even get one person who has done it* (*vasectomy*), *to come out and tell us that it is good or he does not suffer from all the problems we have heard concerning vasectomy*, *I believe it will encourage others to also go and do it*”- (A male participant in FGD).

In interviews with health care providers and the health volunteers revealed that men and women within the communities general showed a low interest in discussions on vasectomy. This apathy created a situation where health providers were reluctant to initiate discussions on vasectomy. This low interest by community members were attributed to the misconceptions and misinformation associated with the procedure.

"*We try to educate the community members on the various family methods but anytime you get to the permanent methods*, *people are reluctant to listen to you*"- (IDI, female CHO).

### Future uncertainty

Clearly, from the study, one of the reasons inhibiting men from accepting vasectomy is the uncertainty about the future. Many respondents raised concerns about their ability to produce children if existing children die or in case of a separation in the present marriage, which will call for remarrying. To community members, the high child mortality, creates the need for couples to be concerned about future contingencies. Another area of concern for men was the possibility of reversing the procedure. The general belief is that if the man decides to remarry in future after vasectomy, it may become difficult to have a child or children with the new wife.

“*Vasectomy is a permanent method*…*if you do it and your children die*, *what do you do*?”- (A male participant in FGD).

In interviews with health care providers, the non-reversibility of the procedure emerged as a well-entrenched theme as one of the reasons why men consider vasectomy as unfavourable family planning method.

*You see*, *because the procedure is permanent*, *the community members are afraid if in future they want to have children but have already done vasectomy*-(IDI, CHO).

In addition, both men and women expressed unease about the potential effect of vasectomy on the sexual faithfulness of their partners. This is because vasectomy was perceived as a guarantee for men to engage in extramarital affairs since they were incapable of impregnating a woman. The nervousness they believe can create problems leading to marital instability.

“*Vasectomy is like giving your man the green light to engage in promiscuous life style*”- (A female participants in FGD).

### Religious constraints

Religion is believed to play a major role in influencing the decision of men to do vasectomy. It was clear from respondents that some religious sects do not approve the use of artificial contraceptives and members stand the risk of being excommunicated if caught using artificial contraceptives. Religious disapproval was even more pronounced when it comes to vasectomy. To some community members’ vasectomy is an infraction against God, which can attract death for a punishment.

“…*so if you go and remove your* ‘*balls*’(*vasectomy*) *within 3–4 years you will die*, *even if you do not die God himself will kill you*”- (A male participant in FGD).

"*The people in this community believe that vasectomy is a sin against God and some church preach against it*"- (IDI, CHV).

Although respondents acknowledged that many religions consider sterilization an infraction against God, some nonetheless suggested that the churches and mosques could be used as platforms to promote the use of contraceptives and vasectomy. They posit that these places of worship reach a wider audience ready to listen to messages from religious authorities without questioning their teachings. Respondents recommended meeting with religious leaders who discourage use of modern contraceptives to address their concerns and to attempt to convince them to accept the use of contraceptives. Religious leaders were perceived to wield so much power in the use of contraceptives over their members and therefore reaching out to them could help increase contraceptives uptake.

“*Family planning services should target the churches and mosques as patrons of this religious denominations strictly follow what their leaders tell them*”- (A male participant in FGD).

### Stigma against vasectomy

Vasectomy is highly stigmatized in the community so men who have undergone the procedure keep it in secret. The belief that the procedure was a form of castration and that it negatively affects men’s sexual functions was widespread, mention in both FGDs and IDIs, and this progressively fueled the stigma on vasectomy. Another factor that affected the stigma was the perception that men who do vasectomy are “women” or "under the control" of their wives. Hence, men were unwilling to lose their authority to their colleague men and women by doing vasectomy. Generally, vasectomized men fear being described in negative and derogatory terms. The stigma therefore makes it difficult for men who have undergone the procedure to advocate for vasectomy uptake.

“*Men who have done vasectomy are people who are controlled by their wives*”- (A man in FGD)

“*My husband has done vasectomy and is still able to satisfy me sexually*…*but in this community*, *they say a lot of negative things about it* (*vasectomy*) *making it difficult for people who have done it* (*vasectomy*) *to come out and say it*”- (A woman in FGD).

"*Men do not want to do vasectomy because people will make fun of them*…*they will say that this man is controlled by his wife*, *therefore he will have no authority in this community*"- (IDI, CHV).

### Perceived alternative to existing male and female contraceptives

It was interesting to note that females in FGD mentioned another form of local male contraceptive method, which was perceived as an alternative to other male and female contraceptive methods. To community members’ two tables of panacin or cafalgen (locally manufactured analgesics) taken by a man before sex could prevent him from impregnating his wife. These drugs according to the female respondents make the sperms watery and therefore incapable of fertilizing an ovum.

“*When I did family planning*, *it was not good for me*, *so now I have my personal way of protecting myself from getting pregnant*, *I use panacin tablets*, *I make my husband to take two tablets before sexual intercourse and this makes the sperms lose their potency*”- (A female in FGD).

“*Many men do their family planning in secret*…. *They take two tablets of cafalgen or two tablets of panacin before any sexual act with their wife and this prevent them from impregnating their wives*”- (A female in FGD).

Men in the community though aware of this perceived method of family planning never mentioned it in FGDs with men until when prompted by the moderators. This practice was widespread among community members and was perceived as a better and more convenient method with minimal adverse reactions as compared to some of the side effects of the female hormonal based methods. The use of condom respondents’ claim takes away the pleasure in having sex (reduces sensation) and therefore prefer to use the locally manufactured analgesics as a contraceptive method.

## Discussion

This study was designed to explore community perceptions about vasectomy and how these perceptions may be affecting vasectomy uptake in Ghana. The findings suggest that women play a very important role in the decision for men to accept vasectomy or not. This is a very important finding in this study because given a spouse’s potential role in the decision-making process, vasectomy promotional efforts should be directed toward women as well as men. The present approach where the emphasis on vasectomy promotional activities is on men may not yield the desire results. It is clear from the study that women were rather more opposed to their husbands going in for vasectomy. Therefore, a man’s decision to do vasectomy revolves around the wife's approval. The perceived impact of vasectomy on the sexual performance of men was a barrier to the uptake of this method. Women generally did not know that vasectomy had no effect on the sexual performance of men. In an earlier study in USA among couples who choose both tubal occlusion and vasectomy, it was reported that woman played key roles in the decision to do vasectomy. Among those who had chosen vasectomy, women were more likely to have discussed the procedure with their partners and to have known a satisfied vasectomy user before making the choice [11].

In this study, virtually all respondents reported hearing negative comments about vasectomy, mostly from friends and community members. These concerns can be dispelled when the correct and accurate information about vasectomy is made available to couples either by health care providers or other vasectomized men. There is therefore evidence that the low use of vasectomy is not entirely because of men’s resistance to the method, but also because of the failure of many health professionals to make information and services available and accessible. Despite efforts to incorporate family planning into the existing Community-based Health Planning and Service, it appears the ability of potential clients to get more accurate and correct information is still a major challenge. In situation where access to correct information is lacking, community members are compelled to rely on misinformation, which are always bothering on myths. In their study in Nairobi, Lynam and colleagues found that 38% of the people said that they learnt about vasectomy from their friends who had undergone vasectomy [12]. Very few indicated medical personnel, parents, and vasectomy counselors as their source of knowledge on vasectomy. Conversely, a study in Pakistan revealed that health workers were significant sources of family planning knowledge [13]. There is therefore the need to provide accurate information to community members. The use of multiple information sources such mass media, home visits and the use of churches and mosques could help in this direction. In addition, the use of couple who have undergone vasectomy to share their experiences about vasectomy would strongly go a long way to disabuse the myths surrounding vasectomy. People generally will choose contraceptive methods that are commonly used in the community and as such deemed socially acceptable [14]. However, in drawing the conclusion that providing accurate information on vasectomy will lead to increase uptake, it is extremely important to acknowledge the supply side because an improved access to information will not necessarily increase the demand for health care if the services are not easily available and if the quality is questionable. Therefore, it is important for family planning providers to ensure that providing information on family planning is supported by the availability of quality services as one of the key determinants of contraceptive uptake and continuous use is clients' satisfaction with the quality of the service [15-17].

In addition, the study revealed that the drawback in people opting for vasectomy is the perceived non-reversibility of the procedure. Vasectomy was a desirable choice when a family considers that they were no longer interested in having children since it was considered a permanent method. The need to have children following the procedure makes the procedure unattractive for couples who are uncertain about future need to have more children. This particular findings supports a previous study that revealed that after 10 years, about 2% of vasectomized men have a reversal operation because of a desire to have children, usually in a new relationship. The chance of a reversal request is increased in men who had a vasectomy at a young age and in those without children [18]. However, this reversal procedure are not without some setbacks as some studies have reported that reversal procedures affect the quality of semen that the man produces though not much has been done in that direction. It appears that the majority of men after vasectomy reversal have reduced semen quality, and sometime additional artificial reproductive techniques are needed to achieve conception [19].

The study further revealed that vasectomy was widely stigmatised with vasectomised men described in derogatory terms. However, given the stigma attached to male sterilisation, even men who have had positive experiences with the procedure might well choose not to reveal or admit this to others in the community, thus limiting the spread of positive stories that are essential to increase acceptance. Family planning services providers should be concerned about this and measures taken to reduce the stigma. The use of innovative ways such as using model couples to promote vasectomy may be more effective. Another strategy that could be used to increase acceptance for vasectomy is to make vasectomy services readily available in communities. Increase knowledge on the nature of the procedure also has the potential of disabusing the minds of people of the perceived effects of vasectomy on both a man’s physical and sexual ability. The women’s attitudes towards vasectomy were also a factor in how active they were in the decision-making process. Women were more often concerned about potential side effects of vasectomy than their male partners. Service providers could strengthen women’s ability to support men’s decision to a greater degree by giving women more information and suggesting ways on how to initiate discussions on reproductive health in a manner that will be acceptable to men. This is especially necessary in places where cultural norms prevent women from initiating discussions on sexual and reproductive health. The use of men-to-men support groups on reproductive health in communities where such gender-based barriers exist may be helpful.

The results from this study indicate that whereas men regarded family planning issues as a responsibility for women, they were not ready to allow women to lead the decision making process. Previous study has reported the possibility of men taking more responsibility for birth control, however, true contraceptive equality remains a challenge [20]. Closely related to this, is a strong historically assumption both in research and in family planning education programmes that men do not readily involve themselves in the reproductive/contraceptive process [21]. Even when individual men might show some interest in being equally involved in the reproductive issues, many of the social structures act as barriers creating a situation where greater reproductive health campaigns are focused almost exclusively on women [22]. This gives a broader perspective to the problem requiring a change in attitude at both the providers and community level. Providers making an assumption and feeling reluctant to discuss contraceptive issues with men reinforces a culture where family planning issues may be regarded as feminine. This in itself will constraint the uptake of contraceptive by male. Gender reshaping and targeting men in family planning services will have the potential to impact men’s behaviour and actions. Some studies have revealed that where men are involved in family planning services it led to greater uptake [23,24]. In involving men in such strategies, it is important to attribute vasectomy to responsibility and care as this may have a far reaching effect and increase vasectomy uptake. Information Education and Communication (IEC) strategies should be designed to associate vasectomy uptake as a masculine responsibility and failure to take up contraceptive methods as a demonstration of irresponsibility on the part of men. Women should be educated to adopt this strategy in initiating inter-partner dialogue on reproductive health and contraceptive use.

One interesting findings in this study was how community members were secretly engaged in the use of a form of a local contraceptive to which many family planning providers were unaware. The use of analgesics (painkillers) as a contraceptive is not evident in literature but was a widely preferred method by community members. Though there may appear to be less adverse reactions associated with the use of such analgesics, there are long-term effects on their health. These drugs are acetaminophen based and the use of acetaminophen has been implicated for liver condition including liver failure. In a study that combined data from 22 specialty medical centers in the United States, acetaminophen-related liver injury was the leading cause of Acute Liver Failure (ALF) for the years 1998 through 2003 [25]. Health care providers should be concerned about this finding and intensify public health education to reduce the use of analgesics as contraceptives.

### Limitations

Although this study fills an important gap in the literature, contributing much needed information for policy decisions, there are a few limitations to be noted. First of all, the FGDs were mostly conducted in the local languages and translated into English, hence some words could have lost their original meanings as a result of the translation. In an attempt to control for possible distortions due to translations, each translation was done by two people and the research team reviewed the translations from the local languages. Nevertheless, given the limitations of such a procedure, little weight was placed on the specific wording or phrasing of responses but the overarching themes. The study was also conducted in two districts both of which are located in southern Ghana, which potentially limits the extent to which the results can be considered applicable outside the specific study context. However, many of the findings are related to similar studies across the world.

## Conclusions

The finding of this study indicates that correct information on vasectomy is still scanty and potential clients base their decisions on myths. Women were very important partners in vasectomy advocacy strategy as they can influence a man’s decision in vasectomy uptake and this should be harnessed in future strategies.

## Competing interests

The authors have declared that no conflict of interest exists.

## Authors’ contributions

PBA, JFP, PT, SA conceived and designed the study: PBA, SFE, EK participated in data collection; PBA, PT-NT, PA did the analysis and writing of the manuscript. All authors read and approved the final manuscript.

## Pre-publication history

The pre-publication history for this paper can be accessed here:

http://www.biomedcentral.com/1472-698X/14/16/prepub
